# Cognitive neuroscience using wearable magnetometer arrays: Non-invasive assessment of language function

**DOI:** 10.1016/j.neuroimage.2018.07.035

**Published:** 2018-11-01

**Authors:** Tim M. Tierney, Niall Holmes, Sofie S. Meyer, Elena Boto, Gillian Roberts, James Leggett, Sarah Buck, Leonardo Duque-Muñoz, Vladimir Litvak, Sven Bestmann, Torsten Baldeweg, Richard Bowtell, Matthew J. Brookes, Gareth R. Barnes

**Affiliations:** a Wellcome Centre for Human Neuroimaging, UCL Institute of Neurology, London, WC1N 3BG, UK; b Sir Peter Mansfield Imaging Centre, School of Physics and Astronomy, University of Nottingham, University Park, Nottingham, NG7 2RD, UK; c UCL Institute of Cognitive Neuroscience, University College London, London, WC1N 3AZ, UK; d Developmental Neurosciences Programme, UCL Great Ormond Street Institute of Child Health, London, WC1N 1EH, UK; e Departamento de Ingeniería Electrónica, Universidad de Antioquia, Medellín, Colombia; f AE&C Research Group, Insituto Tecnológico Metropolitano, Medellín, Colombia

**Keywords:** Optically pumped magnetometers, Language, MEG

## Abstract

Recent work has demonstrated that Optically Pumped Magnetometers (OPMs) can be utilised to create a wearable Magnetoencephalography (MEG) system that is motion robust. In this study, we use this system to map eloquent cortex using a clinically validated language lateralisation paradigm (covert verb generation: 120 trials, ∼10 min total duration) in healthy adults (n = 3). We show that it is possible to lateralise and localise language function on a case by case basis using this system. Specifically, we show that at a sensor and source level we can reliably detect a lateralising beta band (15–30 Hz) desynchronization in all subjects. This is the first study of human cognition using OPMs and not only highlights this technology's utility as tool for (developmental) cognitive neuroscience but also its potential to contribute to surgical planning via mapping of eloquent cortex, especially in young children.

## Highlights

• First cognitive neuroscience experiment using optically pumped magnetometers.
• Language lateralisation is feasible with optically pumped magnetometers.
• Robust within-subject effects at sensor and source level.

## 1 Introduction

Magnetoencephalography (MEG) measures the magnetic fields associated with the electrical activity of the brain, and thus provides a direct quantification of neural population activity. Appropriate mathematical modelling of these fields subsequently allows reconstruction of electrophysiological activity with high temporal and spatial precision (Baillet, 2017; Friston et al., 2008; Hämäläinen et al., 1993). For these reasons MEG has become a useful clinical tool for presurgical evaluation of individuals with epilepsy, particularly for localising the epileptogenic zone via assessment of inter-ictal discharges (Englot et al., 2015; Nakajima et al., 2016; Nissen et al., 2017; Nissen et al., 2016) and for mapping eloquent cortex (Doss et al., 2009; Hashimoto et al., 2017; Hirata et al., 2004, 2010; Pang et al., 2017; Papanicolaou et al., 2014; Wang et al., 2012). MEG's use in this context is highly advantageous as previous research has shown that MEG can change surgical decision making or intracranial EEG placement in up to a third of patients (Sutherling et al., 2008). The role of MEG is even more valuable when one considers that up to 30% of patients may not have an observable lesion on MRI (Duncan et al., 2016), necessitating the use of a functional (as opposed to structural) imaging technique.

As a functional imaging technology MEG has advanced to the stage where it is regularly used in a number of hospitals across Europe, the United States and Japan (Bagič, 2011; De Tiège et al., 2017; Mouthaan et al., 2016; Shiraishi et al., 2012). However in the United States and Europe it is often a few leading hospitals that perform the majority of the scanning. The limited clinical use of MEG outside of these leading centres can be explained by a number of reasons. A conventional MEG scanner is not only expensive to install and maintain but the cryogenic vessel containing the sensors is of a fixed size and generally optimised for the adult head. This means that individuals with smaller heads (i.e. children) whose brains are further from the sensors exhibit lower signal to noise ratio as the signal drops dramatically with distance from the brain (Geselowitz, 1970; Hämäläinen et al., 1993). Furthermore, successful MEG acquisition often requires the subject to keep very still (Stolk et al., 2013) which may not be possible for all subjects.

Here we directly address these issues using a new generation of MEG sensors known as Optically Pumped Magnetometers (Boto et al., 2016). This wearable MEG system does not require cryogenic cooling (reducing cost) and can be placed directly on the scalp (Boto et al., 2016) maximizing signal to noise in paediatric populations. Moreover, recent technical developments allow MEG measurement while a subject is free to move their head (Boto et al., 2018), thus addressing the issue of subject compliance. We show this wearable, and cryogen-free OPM-MEG system can be utilised to map eloquent cortex using a clinically validated language lateralisation paradigm (Liegeois et al., 2002; Pang et al., 2011; Wang et al., 2012). Primarily this highlights how OPMs can be used to provide clinically meaningful information while the head is not constrained to be still, thus better serving non-compliant populations.

**Fig. 1:**
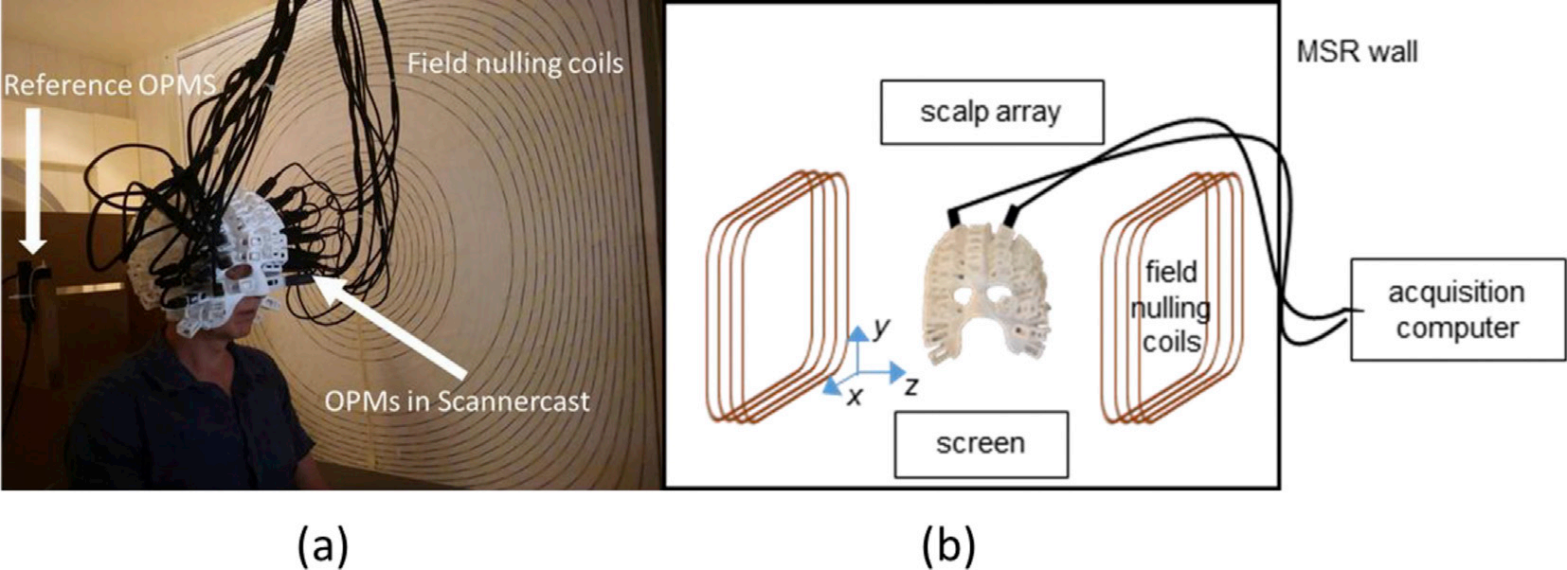
***The OPM setup for studying language lateralisation: real (a) and schematic (b)***. The subject is seated inside the magnetically shielded room wearing the scanner-cast with OPM sensors inserted into slots covering the bilateral aspects of the frontal lobes. The field nulling coils are placed either side of the subject and confer motion robustness to the system by nulling the field over a 40 × 40 × 40 cm^3^ volume within which head movement is tolerated.

To date OPM - MEG has only been used to study primary sensory and motor systems in the human brain and has not been used to study cognitive function (Borna et al., 2017; Boto et al., 2016; Colombo et al., 2016; Kim et al., 2014; Shah and Wakai, 2013; Supek and Aine, 2014; Wyllie et al., 2011; Xia et al., 2006). Here we present a proof-of-concept study demonstrating robust language lateralisation effects at the single subject level using OPM sensors. We focused on a case plus replication study design for two reasons - firstly because within in a clinical setting it is the within subject effects that are of value and secondly because the cost of construction of individualized (MRI derived) OPM scanner-casts limits the number of subjects that can participate. These scanner-casts gave us precise knowledge of sensor orientation and position with respect to the cortex, and also served as scaffolding to support the weight of the sensors and associated wiring (see Fig. 1 and methods for further elaboration). With this demonstration of mapping eloquent cortex and language lateralisation we show that OPMs can in fact be used as a valuable tool for cognitive and clinical neuroscience. In essence this experiment represents an exciting step forward for the use of MEG as it demonstrates the utility of a new generation of wearable MEG sensors for both cognitive and clinical neuroscience with maximal sensitivity throughout the lifespan.

## 2 Methods

### 2.1 Participants

Three healthy subjects (1 female, 2 male) aged 27–50 with normal or corrected-to-normal vision and no history of psychiatric or neurological disorders were recruited to participate in this OPM-MEG study. The research protocol was approved by the University of Nottingham Medical School Research Ethics Committee and written, informed consent was obtained from all participants. The experiments took place at the University of Nottingham. All participants were assessed using the Edinburgh handedness inventory (Oldfield, 1971).

### 2.2 Experimental paradigm

The task presented during the MEG experiment is a variant of a previously validated verb generation task designed to assess language laterality (Liegeois et al., 2002; E. Pang et al., 2011; Wang et al., 2012). Subjects were presented visually with a noun written in the centre of the screen and instructed to think of semantically related verbs without speaking (e.g. if presented with the word “cake”, subjects might think of words such as “bake” or “eat”). They were instructed to continue doing this until the word disappeared from the screen after a 3 s period. Each verb generation period was followed by a baseline period of approximately 2 s where the subject was asked to fixate on a crosshair in the middle of the screen (and no longer think of the noun or verbs related to it). There were a total of 120 trials recorded per subject. All words presented were unique and never repeated within-subject, but noun lists were repeated across subjects after having the order of words randomized. Subjects were not required to respond or speak but reported being able to concentrate and carry out the task throughout the experiment (ca. 10 min duration per subject).

### 2.3 Data acquisition using a novel OPM-MEG system

The bespoke OPM-MEG system (Fig. 1) used here comprised an array of sensors attached to the scalp in a helmet arrangement (the scalp array), a second array of OPM sensors fixed relative to the room (used to characterize background field - the reference array) and a nulling coil system used to remove the effect of the background static (Earth's) magnetic field. Below, each of these components is described in more detail.

#### 2.3.1 OPM sensors

We used an array of 26 OPM on-scalp sensors in addition to four fixed reference OPM sensors placed behind the subjects' heads. The OPMs used are commercially available (http://quspin.com). They have been described in detail elsewhere (Shah and Wakai, 2013) but, in brief, the sensors comprise 3 crucial components a laser (795 nm wavelength), a Rb^87^ vapour cell and a photodiode. The laser induces a transparent steady state in the vapour that allows light to pass through the vapour with minimal absorption of photons and therefore maximal detection of the laser light at the photodiode. As the local magnetic field changes (due to brain activity) the transparency of the gas decreases and less light is detected at the photodiode.

A sinusoidally-oscillating magnetic field, applied using electromagnetic coils which are integrated into the sensor, is used to modulate the magnetic field along two orthogonal axes perpendicular to the laser beam. This allows for the detection of both radial and tangential (to the head) components of the external (neuro-) magnetic field. In this study, only the radial field component was measured. More general overview of the physical principles of OPMs can be found elsewhere (Benumof, 1965; Cohen-Tannoudji et al., 1970; Dupont-Roc et al., 1969; Kastler, 1973; Ledbetter et al., 2008).

The sensors have a bandwidth of approximately 0–130 Hz and dynamic range of approximately ± 1.5 nT. The analogue signal produced by the sensors was sampled at 1000 Hz and quantized with a 16 bit digital acquisition system (National Instruments). All measurements were made inside a magnetically shielded room (MSR) comprising two layers of mu-metal and one of aluminium in order to limit environmental interference.

#### 2.3.2 MRI acquisition and scanner-cast fabrication

Unlike traditional MEG, where the sensors are housed in a fixed array within a cryogenic vessel, the OPM sensors need to be secured relative to the subjects' head. In order to do this, and to provide accurate knowledge of the sensor positions and orientations (necessary for subsequent modelling) custom-made helmets (scanner-casts) were 3D-printed for each individual. To this end a 3D T_1_ weighted magnetic resonance image with the following acquisition parameters was employed: image resolution 1 mm^3^ (1 mm slice thickness) with a field-of view of 256, 256, and 192 mm along the phase (A–P), read (S–I), and partition (R–L; second 3D phase encoding direction) directions respectively. The bandwidth was 425 Hz/pixel, with TE = 2.25 ms TR = 7.96 ms and flip angle of 12°. Partial Fourier (factor 6/8) acquisition was used in each phase-encoded direction. Images were acquired using a Siemens Tim Trio 3T system (Erlangen, Germany). This sequence had been optimised to ensure reasonable contrast of e.g. grey/white matter in the brain, but also minimal distortion around the scalp and face due to susceptibility artefacts at the air/tissue interfaces. Following MRI acquisition, the scanner cast was designed based upon a surface mesh of the scalp/face surface (Boto et al., 2016). The scalp surface was segmented using the unified segmentation approach in SPM12 (Ashburner and Friston, 2005). The ‘isosurface’ function in Matlab was then used to convert the voxel based segmentation to a 3d tessellated mesh. As the mesh and subsequently printed scanner-cast are in the same coordinate space as the MRI image no coregistration is required. Instead the positions are defined based on the centres of the base of the sensor holders with a 6.5 mm offset to account for the sensitive portion of the OPM not being exactly at the base of the sensor. There is some degree of error in this approach based on the tolerance used in the 3D printing which could cause the scanner cast to shift relative to the head if the subject moved quite rapidly. In the worst case this expected to be on the order of 3–5 mm (predominantly due to small rotations of the cast around the anterior-posterior axis). This process of designing custom scanner-casts on a per subject basis currently limits the number of subjects who can participate in out experiment (3 in this case). However, designing these custom scanner-casts is a necessary step to performing source localization of neural activity which has, to date, only been performed with scanner-casts (Boto et al., 2016, 2018). OPM-sensors were then positioned bilaterally to cover the lateral aspects of the frontal and temporal lobes. The setup can be seen in Fig. 1.

#### 2.3.3 Field nulling coils

Subjects were seated comfortably within the shielded room. The shielded room reduced the Earth's magnetic field by a factor of around 2000. However, this still leaves a residual steady field of approximately 25 nT within the room. Although it would have been possible to null this field locally at each sensor using on-board coils; even small head-movements of the subject would be enough to take the sensors outside of their dynamic range ( ±1.5 nT). For this reason we used nulling coils to minimize the Earth's magnetic field around the subject's head (See Fig. 1). Prior to scanning, the currents through these nulling coils (which were designed to generate uniform magnetic fields along three orthogonal directions, as well as the three dominant first-order field gradients) were adjusted so as to minimize the static field as measured by 4 reference OPMs, which were fixed behind the head. These coils are conceptually quite similar to shim coils in MRI in that they produce a homogenous magnetic field (but as close to zero as possible) around the subject's head. The size of this homogenous field is ∼40 × 40 × 40 cm^3^. As long as the subject remains within this volume the sensors should stay within their dynamic range.

### 2.4 Preprocessing

Data were initially down-sampled to 200 Hz, and a power line filter applied to remove 50 Hz mains noise. A second interference source at 77 Hz (this is an aliasing of the modulation field used in the onboard sensor coils which in more recent experiments has been removed by increasing the sampling frequency) was also removed using a stop-band filter. Importantly, since OPMs are configured as magnetometers (as distinct from gradiometers which are used in many cryogenic systems) they are susceptible to increased environmental interference. However this effect can be mitigated by constructing synthetic gradiometers (Boto et al., 2016; Fife et al., 1999); the simplest way to do this involves linearly regressing the signal recorded by the reference array from the signal recorded at the scalp array. The algorithm used in this case involved the creation and inversion (Moore-Penrose pseudoinverse) of a design matrix (X) comprising the four reference sensors. When multiplied by the scalp data (Y) a set of weights (β) were created. These weights defined a linear mixture of the reference sensors that characterized the magnetic interference patterns in the shielded room. This linear mixture was then subtracted from the data to remove the interference. This was done separately on each trial to account for any potential non-stationarity in interference over time.

### 2.5 Spectral responses

**Fig. 2:**
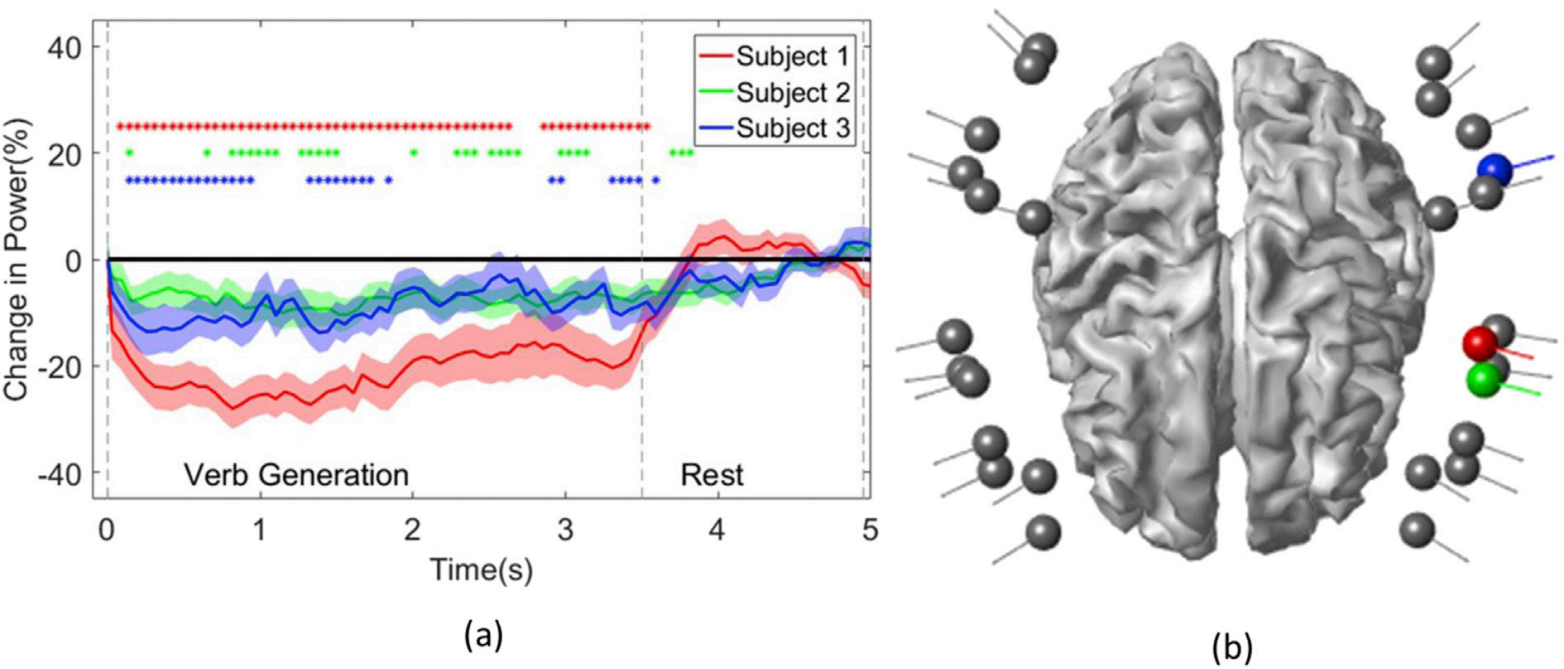
**Sensor level spectral responses (a) and sensor positions (b).** In (a) the greatest sensor level modulation (percentage change relative to rest) of beta band power with respect to the task is presented for each subject. The shading around each line indicates the standard error of the mean as assessed by bootstrapping the average across trials (100 bootstraps). Colour coded asterisks indicate when each subject deviated significantly from rest (p < .05, corrected). The boundary between the task and rest period are indicated by the grey dashed lines. In (b) the sensor positions for performing a language lateralisation experiment on an average subject are plotted relative to the MNI template. The sensors for each subject showing the greatest modulation corresponding to the traces in panel (a) are colour coded accordingly. Note that only 26 sensors were used but more sensors appear in this figure to depict the sensor for each subject that displays the maximum change in beta power.

Previous work on language mapping has showed that a decrease in beta band (15–30 Hz) power is a strong lateralising feature of language in electrophysiological data (Fisher et al., 2008; Spironelli and Angrilli, 2010). We calculate time frequency spectrograms to show how this beta band power changes with our experimental paradigm in sensor space. In each subject we show the sensor with the largest change in beta band power (Fig. 2). Each time course was converted to a percentage deviation from the rest period by normalizing the time series by the average power in the rest period. The percentage power change was then averaged across the task period to identify the largest average change in power. This spectral analysis was performed using the SPM12 implementation of the multi-taper method with a bandwidth of 3 Hz and a smooth time resolution of 1 s. Spectrograms were averaged across trials. We also bootstrapped the mean change in power (sampling with replacement over trials 100 times) to assess the variability of this feature in sensor space.

### 2.6 Source reconstruction

To identify the underlying neural source of the magnetic fields observed with OPMs we used the scalar version of a linear constrained minimum variance beamformer algorithm implemented in the DAISS toolbox for SPM (https://github.com/spm/DAiSS) with source orientation set in the direction of maximal power. We used the Nolte single shell forward model (Nolte, 2003), implemented in SPM12, using the inner-skull boundary derived from the individual T1-weighted MRI.

**Fig. 3:**
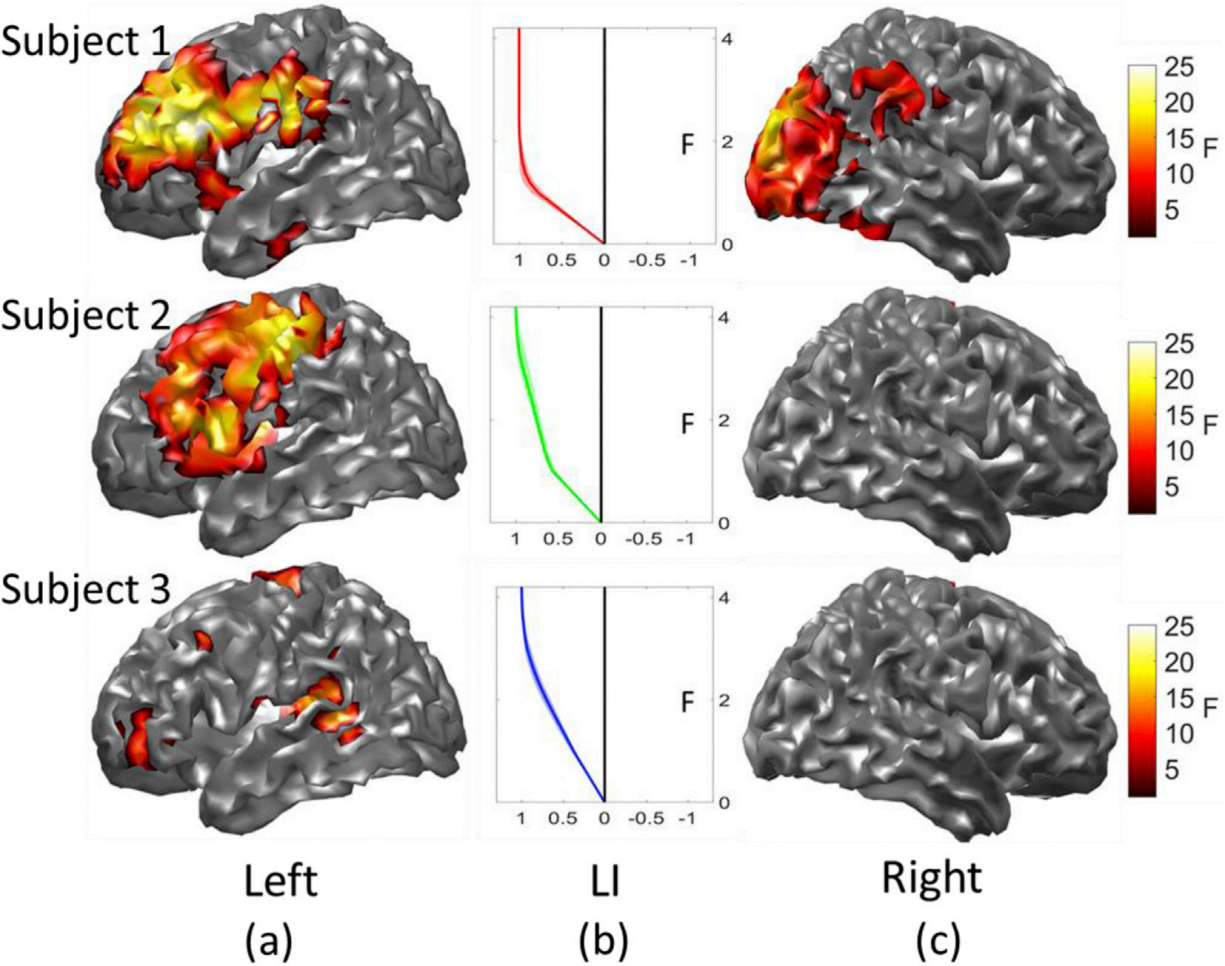
**Language localization (a & c) and lateralisation (b).** Brain Images (a and c, left and right hemisphere) of power change in the beta band for the verb generation task in three subjects. Images are corrected for multiple comparisons using FDR (q < 0.05). In Panel b the LI (Lateralisation Index) displays the relative number of left (positive) hemisphere vs. right (negative) hemisphere voxels in the inferior frontal gyrus as a function of threshold. The localization of the verb generation task is strongly lateralized to the left hemisphere in all 3 subjects as indexed by all LIs tending towards 1 (left) at high thresholds. Confidence intervals (95%) obtained by bootstrapping are indicating by shading surrounding the line plots. The lateralisation index was calculated from thresholds F = 1–15 but is displayed at F = 1–4 as the lateralisation saturates at low thresholds.

The mapping from sensor to source level (i.e. the beamformer weighting) was estimated using a covariance window covering the whole trial and the 15–30 Hz frequency band (5th order bi-directional Butterworth filter). Using these weights, we generated a statistical parametric map (F-statistic) of the power-change in the 15–30 Hz frequency band between the final second of the baseline condition and the first 1 s of the verb generation period. These were then resampled onto a canonical cortical mesh in MNI space (with ∼5 mm spatial sampling) for display purposes (Fig. 3). The resampling procedure was implemented using a nearest neighbor interpolation where the closest voxel (Euclidean distance) to a given vertex governed the intensity of that vertex. Images were then thresholded (whole brain corrected for multiple comparisons) using FDR (q < .05) as implemented in SPM12. To give an indication of the spatial resolution and within subject variability of this dataset we performed a bootstrapping procedure (50 bootstraps across trials) on the peak activity in the inferior frontal gyrus (a key region for language) to construct confidence volumes (Fuchs et al., 2004). This procedure resulted in 50 difference localisations. The 95% confidence volume was approximated as twice the standard deviation of the bootstrapped localizations (in MNI coordinates).

### 2.7 Lateralisation indices

A number of different metrics have been used to assess laterality in the functional imaging literature each having different strengths and weaknesses (Wilke and Lidzba, 2007). In this study we used a region of interest approach where we calculated the relative supra-threshold voxel count in the inferior frontal gyrus (where we also calculate confidence volumes) of both hemispheres (Liegeois et al., 2002). This was calculated explicitly as in Eq (1) across statistical height thresholds (F = 1–15).

(1) $$LI=\;\frac{\sum voxels_{left}-\;\sum voxels_{right}}{\sum voxels_{left}+\;\sum voxels_{right}}$$

Where $\sum voxels_{left}$ and $\sum voxels_{right}$ count the number of voxels above a given threshold. This metric gives a value in between the range of −1 and 1 with positive values indicating left lateralisation. To assess the variability in this metric we bootstrapped the relative power change across trials (100 bootstraps) resulting in 100 different source localisations and 100 different lateralisation indices. This allowed us to quantify the uncertainty in our lateralisation indices with 95% confidence intervals across thresholds.

### 2.8 Source level time courses

We reconstructed the electrophysiological time series for each subject at the location of maximum beta power change. The time course was constructed using the location-specific beamformer weights derived from the source reconstruction stage.

### 2.9 Software

All analysis was carried out using SPM12 http://www.fil.ion.ucl.ac.uk/spm/and the DAISS toolbox (https://github.com/spm/DAiSS).

## 3 Results

### 3.1 Sensor level response

Fig. 2 shows the average relative change in beta band power over the course of the task for the sensor with the largest beta band change for each subject. During the task period a drop in beta band power is noted that then returns to baseline during the rest period. In terms of laterality all 3 sensors with maximum change are situated over the left hemisphere. However the exact source of the neural activity is still uncertain and motivates a source space analysis.

### 3.2 Source reconstruction

Fig. 3 shows the statistical maps of electrical power change in the beta-band over the left (a) and right (c) hemispheres. All three subjects display a highly left-lateralized source localization as indexed by the laterality indices (LI, panel b) which rapidly tends towards 1 as a function of statistical threshold. This is to be expected as all subjects are unlikely (∼5% chance) to be right hemisphere dominant for language based on their handedness scores (Knecht, 2000). In terms of consistency all subjects displayed activity in the left inferior frontal gyrus, left middle frontal gyrus and left postcentral gyrus (see Table 1 for MNI coordinates and statistical values).

**Table 1:** **Location of local maxima in 3 subjects**. Labels are defined using the AAL atlas.

		MNI Coordinate			
		x	y	z	F-statistic
**Subject 1**	*Left Inferior Frontal*	−53.26	17.3	19.03	26.2
	*Left Postcentral*	−59.13	−11	30.78	24.08
	*Left Middle Frontal*	−32.49	43.85	29.29	23.21
**Subject 2**	*Left Inferior Frontal*	−48.64	13.99	9.36	19.49
	*Left Postcentral*	−43.08	−16.52	40.54	21.08
	*Left Middle Frontal*	−46.4	21.36	32.9	16.06
**Subject 3**	*Left Inferior Frontal*	−41.2	43.25	−5.86	11.19
	*Left Postcentral*	−55.51	−14.6	14.9	10.9
	*Left Middle Frontal*	−43.15	16.17	43.28	12.24

Having bootstrapped the localization in the inferior frontal gyrus the 95% confidence intervals (axis length of confidence ellipse) for subject 1, 2 and 3 in the x, y and z direction were as follows: (2.4 mm, 1.06 mm, and 8.04 mm), (7.44 m, 1.56 mm, 2.2 mm) and (1.64 mm, 1.40 mm, 7.4 mm). A conservative estimate of the spatial resolution (or within subject variability in peak localization) would be to take the maximum of these numbers which is between 7 and 8 mm per subject. This is comparable to previous estimates of MEG spatial resolution (Brookes et al., 2010; Hedrich et al., 2017).

### 3.3 Source level time courses

**Fig. 4:**
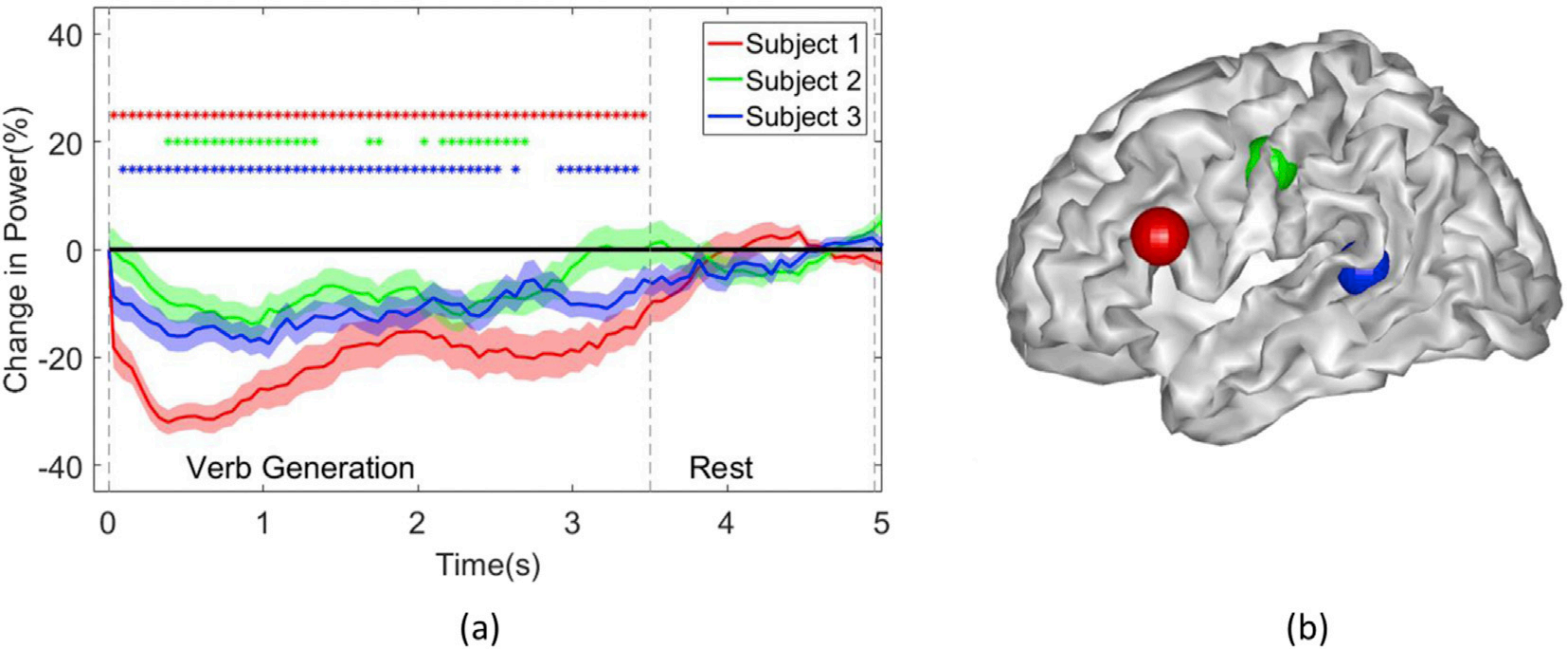
**Source level time-course of maximal beta power change.** In (a) the maximum beta band power change (%) from baseline is plotted over the course of the task for each subject. Shading indicates standard error as assessed by bootstrapping (100 bootstraps over trials). Colour coded asterisks indicate when each subject deviate significantly from rest (p < .05, corrected). In (b) the location of these maximal beta band power changes (all left hemisphere) are indicated by the spheres imbedded in the surface render of the MNI template.

We now provide examples of the electrophysiological time series upon which the statistical maps in Fig. 3 are based. In Fig. 4(a) the maximum beta band power change for each subject is plotted. A beta desynchronization is consistently observed during task period across all subjects and returns to baseline during the rest period for all subjects. The location of this effect is observable in Fig. 4(b).

## 4 Discussion

We have demonstrated that a wearable OPM-MEG system can be used to perform a clinically important language lateralisation paradigm in healthy adults on a case by case basis. The findings across the 3 subjects are consistent with previous literature both in fMRI (Binder et al., 2009; McGuire et al., 1996a, b; Price, 2010, 2012) and MEG (Fisher et al., 2008; Hirata et al., 2010). This is also the first study to examine human cognition using OPMs. Importantly, the array is wearable and motion robust (Boto et al., 2018) meaning that it could have direct and practical implications in clinical paediatric assessment or indeed developmental neuroscience.

Over the last 25 years neuroimaging has provided a wealth of information on the functional neuroanatomy of speech and language, furthering our understanding of cognition (Price, 2012) and providing direct clinical applications such as non-invasive mapping of eloquent cortex (Fisher et al., 2008; Mouthaan et al., 2016; E. Pang et al., 2011; Wang et al., 2012). However, despite all that we have learned we still know relatively little of the neurodevelopment of human language and more generally cognition in young children. Furthermore, non-invasive clinical applications such as presurgical assessment between the ages of 2–8 years old are fraught with movement and compliance issues in all neuroimaging modalities. Although, some important algorithms and technical developments have been utilised to mitigate these issues in both MEG and fMRI (Wehner et al., 2008; Zaitsev et al., 2006). Specifically, with regards to cryogenic MEG, movement of the head away from the sensors results in signal loss that can never be recovered (although mitigated through extrapolation) leading to a deleterious effect on data quality.

By using a wearable OPM-MEG system we can guarantee maximal signal at all times which will be a key benefit when utilised with paediatric populations where compliance can be an issue. We expect these advantages to be magnified in studies where the head-motion is task-dependent such as motor control experiments or the production of overt speech. For example, it has been suggested that the production of overt speech is necessary when performing language paradigms in children as it increases yield of pre-surgical mapping (Croft et al., 2013). As such, head motion is unavoidable and a technique that is robust to the impact of motion-such as the OPM-MEG (Boto et al., 2018)- is essential for presurgical mapping of eloquent cortex and tracking neurodevelopment in children.

As we intend to develop OPM-MEG into a useful tool for clinical neuroscience an examination inter and intra-subject variability of this technology is of interest. We have shown significant within subject effects at the sensor and source level (Fig. 2, Fig. 4). The spatial localisations within subject are also robust as demonstrated by confidence volumes (5–10 mm) which are comparable with previous MEG studies of spatial resolution (Brookes et al., 2010; Hedrich et al., 2017) and lastly, the resulting lateralisation indices at the source level are also consistent within subject (as verified by the bootstrapped confidence intervals: Fig. 3).

However, there are spatial differences between participants in the exact location of the beta desynchronization. For instance subject 1, displays a large decrease in beta band power in occipital regions whereas as subject 2 and 3 do not. As we did not discuss with our participants what cognitive strategy they used for completing the task it is feasible that this contributes to the inter subject variability in spatial localization. This has previously been extensively documented In the context of the language network (Kherif et al., 2009; Seghier and Price, 2016, 2018). However considering, all subjects consistently activated key nodes (inferior frontal gyrus, middle frontal gyrus and postcentral gyrus) of the language network (Fisher et al., 2008; Hirata et al., 2004, 2010; Price, 2012) we believe both the inter and intra-subject variability is sufficiently low to pursue studies examining how we may use OPM-MEG in clinical context where small intra-subject variability is vital.

The usability of OPM-MEG is currently compromised by the size of the individualized helmet that is used to house the sensors and provide support for the weight of the associated cabling. Currently, this is necessary in order to ensure accurate knowledge of sensor position relative to the brain while providing sufficient support for the sensors. Unfortunately, this currently limits the number of participants that can take part in the study. However, the sensors are rapidly decreasing in size to dimensions comparable to an EEG electrode (Alem et al., 2014). With the advent of smaller sensors we should be able to create more lightweight OPM arrays in the form of flexible, reusable helmets. The relative sensor positions and orientations of these arrays could then be estimated post-hoc by using the nulling coils (Fig. 1) to set up predictable gradient fields. Subsequently, advanced post processing (López et al., 2012) could be used to determine the precise array location with respect to the cortical surface. With these advances the use of OPMs should no longer be restricted to studies with a few participants but could be extended to much greater sample sizes.

There are also still some practical issues in the OPM implementation-such as cross-talk between nearby sensors (the degree to which the modulation field of one sensor may change the gain of a nearby sensor). This is currently of little consequence for our studies as the distance between our sensors results in cross-talk of less than 5% (Boto et al., 2018). However, this effect will need to be examined in more detail in order to push the spatial resolution of OPM-MEG to the extent of previous cryogen based MEG systems (Bonaiuto et al., 2017; Meyer et al., 2017; Troebinger et al., 2014).

We have demonstrated a wearable MEG system that can be used for robust single-subject studies of language lateralisation. As the system is wearable, cryogen free and rapidly reducing in size (allowing for potentially flexible and reusable helmets) we expect the running costs of MEG to reduce dramatically. The ability to perform standard cognitive paradigms with these arrays, coupled with their wearability and motion robustness opens exciting new avenues both in clinical and developmental neuroscience.
